# How to Sync to the Beat of a Persistent Fractal Metronome without Falling Off the Treadmill?

**DOI:** 10.1371/journal.pone.0134148

**Published:** 2015-07-31

**Authors:** Melvyn Roerdink, Andreas Daffertshofer, Vivien Marmelat, Peter J. Beek

**Affiliations:** 1 MOVE Research Institute Amsterdam, Department of Human Movement Sciences, Faculty of Behavioural and Movement Sciences, VU University Amsterdam, Amsterdam, The Netherlands; 2 Movement to Health Laboratory, Euromov, University of Montpellier, Montpellier, France; Purdue University, UNITED STATES

## Abstract

In rehabilitation, rhythmic acoustic cues are often used to improve gait. However, stride-time fluctuations become anti-persistent with such pacing, thereby deviating from the characteristic persistent long-range correlations in stride times of self-paced walking healthy adults. Recent studies therefore experimented with metronomes with persistence in interbeat intervals and successfully evoked persistent stride-time fluctuations. The objective of this study was to examine how participants couple their gait to a persistent metronome, evoking persistently longer or shorter stride times over multiple consecutive strides, without wandering off the treadmill. Twelve healthy participants walked on a treadmill in self-paced, isochronously paced and non-isochronously paced conditions, the latter with anti-persistent, uncorrelated and persistent correlations in interbeat intervals. Stride-to-stride fluctuations of stride times, stride lengths and stride speeds were assessed with detrended fluctuation analysis, in conjunction with an examination of the coupling between stride times and stride lengths. Stride-speed fluctuations were anti-persistent for all conditions. Stride-time and stride-length fluctuations were persistent for self-paced walking and anti-persistent for isochronous pacing. Both stride times and stride lengths changed from anti-persistence to persistence over the four non-isochronous metronome conditions, accompanied by an increasingly stronger coupling between these gait parameters, with peak values for the persistent metronomes. These results revealed that participants were able to follow the beat of a persistent metronome without falling off the treadmill by strongly coupling stride-length fluctuations to the stride-time fluctuations elicited by persistent metronomes, so as to prevent large positional displacements along the treadmill. For self-paced walking, in contrast, this coupling was very weak. In combination, these results challenge the premise that persistent metronomes in gait rehabilitation would evoke stride-to-stride dynamics reminiscent of self-paced walking healthy adults. Future studies are recommended to include an analysis of the interrelation between stride times and stride lengths in addition to the correlational structure of either one in isolation.

## Introduction

Estimation of the scaling behavior of serial-lag correlations in time series of gait variables, like stride times, stride lengths, and stride speeds, has become common practice in human gait research [1–20]. In the majority of papers the correlational structure of stride-to-stride fluctuations is quantified using detrended fluctuation analysis (DFA, [21]), which assesses the relation between the magnitude *F* of stride-to-stride fluctuations and the time interval *n* over which these fluctuations are observed. When the relation obeys the power-law *F*(*n*) ~ *n* ^*α*^ the correlational structure of the stride-to-stride fluctuations is scale-free or fractal. If in such scale-free or fractal time series deviations are statistically more likely to be followed by subsequent deviations in the same direction, then a time series exhibits statistical persistence, as deviations persist over subsequent data points. If deviations are statistically more likely to be followed by subsequent deviations in the opposite direction, then a time series exhibits statistical anti-persistence. Subsequent deviations are uncorrelated if deviations are randomly followed by deviations in the same or opposite direction. The value of the DFA scaling exponent α indicates statistical persistence (α > 0.5) or anti-persistence (α < 0.5) in a time series, as well as a lack thereof (α ≈ 0.5). During unconstrained overground walking [17], stride times, stride lengths, and stride speeds typically exhibit statistical persistence (α > 0.5), whereas elderly and patients with central nervous system disorders may lose statistical persistence in certain gait variables in that deviations become either uncorrelated (α ≈ 0.5) or anti-persistent (α < 0.5) (e.g., [22–24]).

Two interpretations of such findings have been suggested in literature. The first interpretation holds that statistically persistent fluctuations are an identifying feature of healthy physiological functioning (“healthy complexity”), whereas uncorrelated and anti-persistent fluctuations indicate aging, disease and pathology [22]. This “pathological breakdown of complexity” with aging and disease is thought to reduce the adaptive capabilities of an individual (e.g., [25–28]) and has been documented for a wide variety of physiological functions, including heart rate dynamics, respiration, postural control, and walking. In a similar vein, the theory of optimal movement variability, proposed by Stergiou and colleagues [29–31], postulates that there is an optimal level of complexity associated with healthy and proficient behavior (see also [32]). Gait therapies developed in accordance with these frameworks should aim to restore optimal gait variability, operationalized as statistically persistent stride-to-stride fluctuations [7, 8, 10, 14, 20, 31].

Dingwell and Cusumano [3] proposed an alternative interpretation by taking α to reflect the tightness of control over gait variables: gait variables that are not tightly regulated exhibit statistical persistence (i.e., deviations persist over multiple consecutive strides) while deviations in tightly controlled gait variables are anti-persistent (i.e., deviations are immediately corrected in subsequent strides). This interpretation was inspired by the observation that walking in time to a metronome, which requires that stride times are tightly regulated, changed the correlational structure of stride times qualitatively from persistent to anti-persistent [2, 6, 11, 16–19]. As regards interpreting anti-persistence as a pathological marker of breakdown of healthy complexity, Dingwell and Cusumano [3] proposed that the locomotor system could not be ‘healthy’ by producing persistent fluctuations when self-paced and–by the same token–‘unhealthy’ by producing anti-persistent fluctuations with isochronous pacing. They suggested that the diminution of the fractal structure with acoustic pacing and with pathology may reflect an increase in stride-to-stride gait control (i.e., ‘cautious gait’). For motorized treadmill walking, Dingwell and Cusumano [3] likewise hypothesized that participants strictly regulate stride speeds to prevent walking off the treadmill. They indeed found that stride speeds became anti-persistent for treadmill walking, while stride times and stride lengths remained persistent [3]. For metronome-paced treadmill walking, one would accordingly expect anti-persistence in stride times (coupling to the beat), anti-persistence in stride speeds (controlling position on the belt) and, because stride length is the product of stride time and stride speed, also anti-persistence for stride lengths. Terrier and Dériaz [18] recently found that this was indeed the case. In these examples, anti-persistence was observed only for the gait variables that required tight control, such that deviations in the controlled variables were rapidly countered [3].

Inspired by the observation that stride-time dynamics evoked by an isochronous metronome typically results in anti-persistent stride-to-stride fluctuations [2, 6, 11, 16–19], several recent studies have explored the use of non-isochronous metronomes with persistence in interbeat intervals [8, 10, 11, 20]. The premise was that by using persistent metronomes, evoked stride-time dynamics would likely more closely resemble that of self-paced walking healthy participants (exhibiting persistent stride-to-stride fluctuations), which agrees with notions of optimal movement variability [29–31] and pathological breakdown of complexity [22, 25–28]. Of specific interest is the study by Marmelat and colleagues [11], in which non-isochronous metronomes were used that differed in terms of the correlational structure in interbeat intervals. Participants were instructed to walk to the beat of non-isochronous metronomes, with anti-persistent (α ≈ 0.2), uncorrelated (α ≈ 0.5) or persistent (α ≈ 0.6 and α ≈ 0.9) correlational structures in interbeat intervals. Similar to uncued walking, persistence in stride times was observed when the non-isochronous metronome contained persistent correlations, which led to the conclusion that “the presence of long-range correlations in auditory cues enabled participants to maintain their ‘normal’, fractal gait pattern” ([11], page 8, second column, line 12; see also [8, 10, 14, 20]).

These qualitatively similar scaling exponents α of stride times for the self-paced walking condition and the non-isochronous metronome condition with α ≈ 0.9 is a remarkable finding because persistence in stride times implies that deviations in stride times persist over multiple consecutive strides during treadmill walking. Without concomitant (coupled) adjustments in stride lengths and/or stride speeds, this would result in a considerable fore-after displacement along the treadmill. That is, with multiple consecutive shorter-than-average stride times (i.e., higher cadence), participants would displace towards the front edge of the treadmill. Likewise, with multiple consecutive longer-than-average stride times, participants would displace backwards. In either case, participants would wander off the relatively short treadmill belt when paced by a persistent metronome.

This wandering off the treadmill, however, did not occur in the experiment of Marmelat et al. [11], which suggests that the observed persistence in stride-time deviations may have been cancelled out by concomitant persistent changes in stride lengths. In line with the hypothesis and findings of Dingwell and Cusumano [3] such a coupling between stride times and stride lengths would indeed allow for persistence in those variables while simultaneously controlling the fore-after displacement along the treadmill, yielding anti-persistence in stride speeds (see also [18]). However, compared to self-paced treadmill walking (as studied in [3]), one may expect the coupling between the two gait variables to be much stronger for treadmill walking paced by persistent metronomes than for the other pacing conditions given the inherent risks involved with following a metronome with persistent interbeat intervals (i.e., longer/shorter interbeat intervals are likely to be followed by multiple consecutive interbeat intervals in the same direction).

The purpose of the present study was to gain more insight into the gait dynamics for isochronously and non-isochronously paced treadmill walking. We reanalyzed the data of Marmelat et al. [11] and evaluated the DFA scaling exponent α not only for stride times but also for stride lengths and stride speeds. For isochronous pacing, all gait parameters were expected to become anti-persistent (cf. [18]). To prevent wandering off the treadmill with non-isochronous metronomes, anti-persistence for stride speeds was expected in all conditions and manifested by simultaneously regulating both stride times and stride lengths (i.e., a positive correlation between stride-time and stride-length time series). Because Marmelat et al. [11] found that the DFA scaling exponent α of stride times largely matched the DFA scaling exponent α of the non-isochronous metronomes, we expected an increase in α for both stride times and stride lengths from anti-persistence to persistence over the four non-isochronous metronome conditions (i.e., α ≈ 0.2, α ≈ 0.5, α ≈ 0.6, and α ≈ 0.9). Finally, given that the inherent risk of wandering off the treadmill increases over these four metronome conditions, we expected an increasingly stronger coupling between stride times and stride lengths, with peak correlation values for the persistent metronomes, in order to prevent large positional displacements along the treadmill.

Answering the question how participants couple their gait to the beat of a persistent metronome without falling off the treadmill may help to evaluate the premise that by using persistent metronomes in gait rehabilitation stride-to-stride dynamics would more closely resemble that of self-paced walking healthy participants.

## Materials and Methods

### Participants

Twelve volunteers (five female, age 28 ± 6 years, mean ± *SD*) participated in the experiment. All participants were healthy and none had neuromuscular disorders or injuries at the time of study.

### Ethics Statement

The research met all applicable standards for the ethics of experimentation and was approved by the Ethics Committee of the Faculty of Human Movement Sciences of VU University Amsterdam. Participants provided written informed consent prior to the experiment.

### Experimental setup

Participants walked on a motorized treadmill in which a single large force platform was embedded (ForceLink, Culemborg, The Netherlands). Computer-generated rhythmic auditory stimuli (pitch 600 Hz) were administered through earphones (right ear), to pace the right heel strikes. Metronome beats and force platform data were sampled simultaneously at 300 Hz.

### Experimental procedure

All details about experimental procedures can be found in the original report by Marmelat et al. [11]. We here briefly sketch the part that was reanalyzed. First, each participant’s preferred treadmill walking speed was determined (4.15 ± 0.44 km/h), which was used for the subsequently performed uncued treadmill walking condition (UC) as well as for the acoustically paced treadmill walking conditions with an isochronous metronome containing equidistant interbeat intervals (ISO) and the four non-isochronous metronomes containing interbeat interval sequences with distinct scaling exponents (α ≈ 0.2, α ≈ 0.5, α ≈ 0.6 and α ≈ 0.9). The order of these five pacing conditions was randomized. Participants were instructed to synchronize the right heel strikes with the beats of the metronome and to maintain this synchronization as accurately as possible while keeping a natural and relaxed gait in case the metronome would present irregularity. Participants received gait-tailored metronome sequences, with mean interbeat intervals being equal to their mean stride times observed for uncued treadmill walking (UC condition). The coefficient of variation over interbeat intervals was fixed at 1% for all non-isochronous metronomes. Every condition lasted six minutes in order to be able to determine scaling exponents with excellent within-day test-retest reliability (ICC > 0.9, [12]).

### Data analysis

Right stride times, stride lengths and stride speeds time series were derived from online determined right heel strike events [33] and associated anterior-posterior center-of-pressure positions [34]. Specifically, stride times (ST in s) were defined as the time intervals between consecutive right heel strikes events. Stride lengths (SL in m) were derived by multiplying these stride times with the belt speed (in m/s), while correcting for spatial separation in consecutive right heel-strike positions on the treadmill surface [34]. Finally, stride speeds (SS in m/s) were calculated as the ratio of stride length over stride time for each stride cycle [3]. To prevent transient effects related to the fact that a number of strides are required to reach synchronization with the beat (see [35]), the first 10 strides were eliminated. The central 256 ST, SL and SS data points from the remaining strides were used for further analyses. These data are presented as Supporting Information (S1 Dataset).

The following set of complementary outcome measures was determined: DFA scaling exponent α, maximal absolute anterior-posterior displacement along the treadmill and the coupling between ST and SL. As regards the first outcome measure, the mean square roots of linearly detrended residuals of the integrated mean-centered ST, SL and SS time series (*F*(*n*)) were calculated for 50 exponentially spaced non-overlapping intervals of *n* data points. The DFA scaling exponent α of ST, SL and SS was then estimated by the taking the slope of the log-log plot of *F*(*n*) against *n*-values (i.e., from *n* = 10 to *n* = 128 samples) [21]. To obtain the second outcome measure, for each trial the maximal absolute value of the cumulative sum of mean-centered SS time series was determined, reflecting the maximal displacement along the treadmill. For the final outcome measure–the coupling strength between ST and SL time series–we determined the correlation between ST and SL time series. Positive correlations were expected for all conditions implying that stride lengths and stride times vary over time in a similar way, with the strongest correlations for the persistent fractal metronome conditions.

### Surrogate data

We hypothesized that the assumed anti-persistence in SS would be manifested by simultaneous regulation of ST and SL, in particular for persistent fractal metronome conditions. In addition to the correlation between ST and SL time series, surrogate data were employed to verify or falsify this general hypothesis. To this end, we randomized the phases after Fourier transformation of original time series, after which the inverse Fourier transform was applied, yielding so-called phase-randomized surrogate time series [36]. Due to the preservation of the power spectra as well as the auto-correlation properties in phase-randomized surrogates (cf. [36]), the statistical persistence in phase-randomized and original time series is similar by definition, and thus the associated DFA scaling exponents α (within computational limits). In the present study, 20 phase-randomized surrogate time series were generated for each original ST and SL time series, either *independently* of each other (yielding phase-randomized surrogates) or *simultaneously* for ST and SL in exactly the same manner (yielding cross-correlated phase-randomized surrogates). This latter procedure was of special importance for testing the general hypothesis because it not only preserves the statistical persistence of ST and SL individually, but also the cross-correlation between them [3]. Thus, while by definition DFA scaling exponents α of phase-randomized surrogates and cross-correlated phase-randomized surrogates are expected to match the DFA scaling exponents α of the original ST and SL time series, only the DFA scaling exponents α of the cross-correlated phase-randomized surrogates are expected to match the DFA scaling exponents α for original SS time series in all conditions (bear in mind that surrogate SS time series are calculated as the ratio of surrogate SL over surrogate ST time series).

### Statistics

To corroborate statistical persistence or anti-persistence in ST, SL and SS time series, the respective DFA scaling exponents α were subjected to one-sample *t*-tests against 0.5 (reflecting uncorrelated noise). All outcome measures (i.e., maximal displacement, DFA scaling exponent α for ST, SL and SS time series and the correlation between ST and SL) were examined using a repeated-measures ANOVA with Condition as within-subject factor (6 levels: UC, ISO, α_0.2_, α_0.5_, α_0.6_ and α_0.9_). Degrees of freedom were adjusted with the Huynh-Feldt method if the assumption of sphericity was violated. Effects were labeled significant if *p* < 0.05 and effect sizes (*ES*) were expressed as partial *η* ^2^. Post hoc analysis entailed two-tailed paired-samples *t*-tests.

## Results

**Fig 1:**
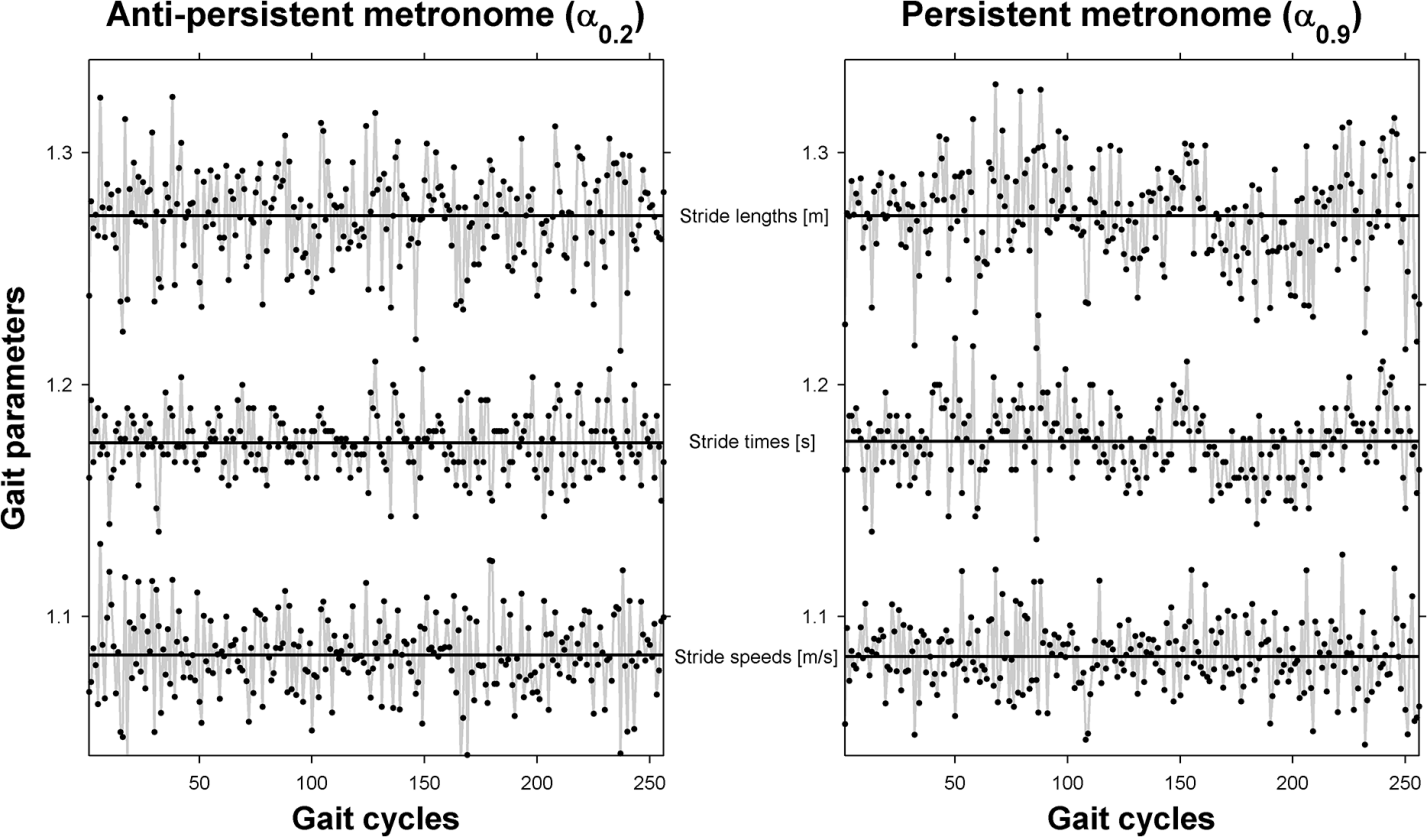
Examples of stride-to-stride fluctuations. Representative examples of stride-time, stride-length and stride-speed time series for two non-isochronous metronome conditions with anti-persistence (α_0.2_, left panel) and persistence (α_0.9_, right panel) in interbeat intervals. The correlation between stride-times and stride-length time series was positive, and much stronger for the persistent (*r* = 0.61, right panel) than for the anti-persistent (*r* = 0.36, left panel) metronome condition.

Fig 1 depicts representative ST, SL and SS time series of a single participant for two non-isochronous pacing conditions with α_0.2_ (left panel) and α_0.9_ (right panel). As can be appreciated from this figure, the DFA scaling exponents α of ST and SL qualitatively mimicked the anti-persistent and persistent nature of the metronome (ST: α = 0.232, SL: α = 0.317 and ST: α = 0.786, SL: α = 0.692, respectively), whereas SS time series exhibited anti-persistence for both cases (α = 0.289 and α = 0.387, respectively). As reflected by positive correlations between ST and SL, which were stronger for the persistent (*r* = 0.61) than for the anti-persistent metronome (*r* = 0.36), this participant apparently strongly coupled SL fluctuations to the ST fluctuations elicited by the persistent metronome, in order to preserve his or her walking position along the treadmill.

### Scaling exponents and maximal displacement

**Fig 2:**
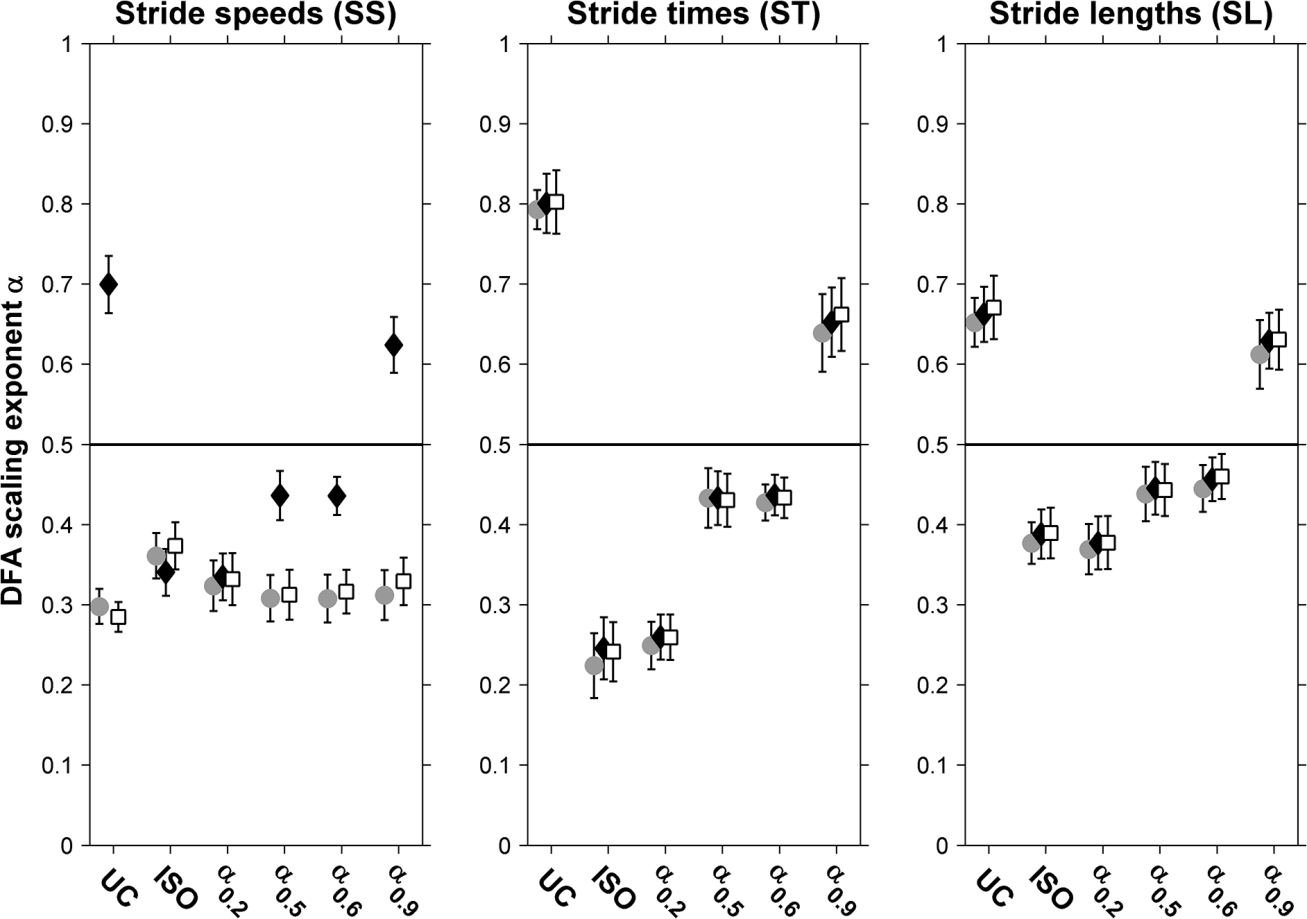
Scaling exponents for original and surrogate time series. DFA scaling exponents α for stride speeds (left panel), stride times (center panel) and stride lengths (right panel) for uncued treadmill walking (UC) and isochronous (ISO) and non-isochronous (α_0.2_, α_0.5_, α_0.6_, α_0.9_) metronome conditions. DFA scaling exponents α are given for original time series (gray circles) as well as for phase-randomized and cross-correlated phase-randomized surrogates (black diamonds and white squares, respectively).

Fig 2 depicts the DFA scaling exponent α of original and surrogate time series for SS, ST and SL for each condition. One-sample *t*-tests against 0.5 revealed that the DFA scaling exponent α for SS was, as expected, anti-persistent for all conditions (all *t*(11) < -4.8, all *p* < 0.001; see Fig 2A). For ST and SL time series, in contrast, the correlational structure varied qualitatively over conditions. One-sample *t*-tests against 0.5 showed that for uncued treadmill walking the stride-to-stride fluctuations were persistent (ST: α = 0.793 ± 0.024, *t*(11) = 12.0, *p* < 0.001; SL: α = 0.652 ± 0.030, *t*(11) = 5.0, *p* < 0.001). With isochronous pacing, stride-to-stride fluctuations changed qualitatively from persistent to anti-persistent for both ST (α = 0.224 ± 0.040; *t*(11) = -6.8, *p* < 0.001) and SL (α = 0.377 ± 0.026; *t*(11) = -4.7, *p* < 0.001). With non-isochronous pacing, α changed as expected qualitatively and significantly from anti-persistent for α_0.2_ to persistent for α_0.9_ for both ST (α_0.2_: 0.249 ± 0.030, *t*(11) = -8.4, *p* < 0.001; α_0.9_: 0.639 ± 0.048, *t*(11) = 2.9, *p* = 0.015) and SL (α_0.2_: 0.369 ± 0.031, *t*(11) = -4.2, *p* = 0.002; α_0.9_: 0.612 ± 0.043, *t*(11) = 2.6, *p* = 0.024) alike. For α_0.5_, no differences from 0.5 were observed for α of ST (α_0.5_: 0.433 ± 0.037, *t*(11) = -1.8, *p* = 0.099) and SL (α_0.5_: 0.438 ± 0.034, *t*(11) = -1.8, *p* = 0.096). For α_0.6_, α was significantly smaller than 0.5 for ST (α_0.6_: 0.428 ± 0.022, *t*(11) = -3.2, *p* = 0.008) whereas α did not differ from 0.5 for SL (α_0.6_: 0.445 ± 0.029, *t*(11) = -1.9, *p* = 0.086).

**Fig 3:**
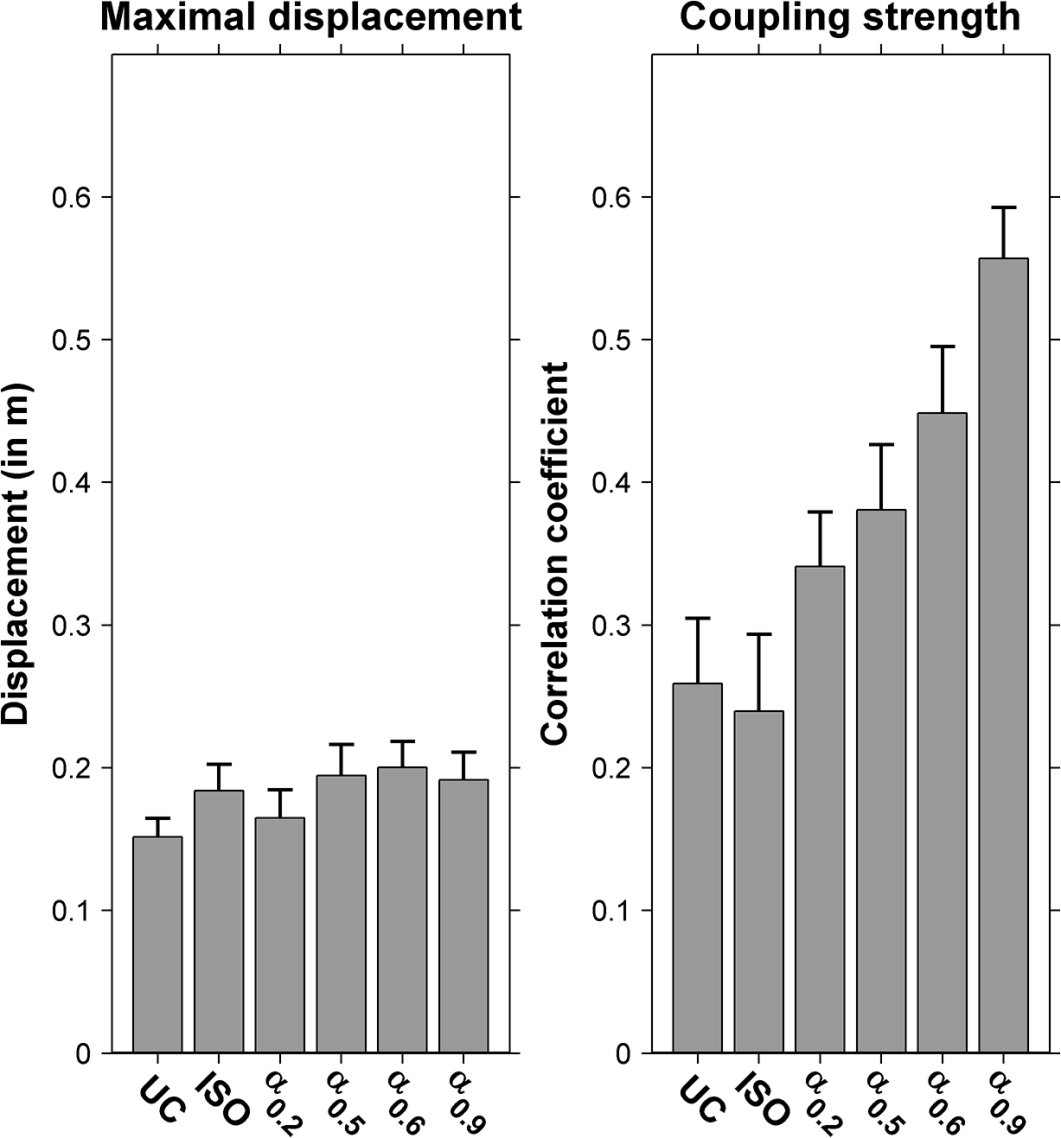
Maximal displacement and coupling strength. Results of maximal displacement along the treadmill (left panel) and the coupling strength between stride times and stride lengths (right panel) for uncued treadmill walking (UC) and isochronous (ISO) and non-isochronous (α_0.2_, α_0.5_, α_0.6_, α_0.9_) metronome conditions.

The repeated-measures ANOVA revealed that the DFA scaling exponent α for SS did not vary systematically over conditions (0.318 ± 0.018; *F*(5, 55) = 0.81, *p* = 0.545, *ES* = 0.069). Likewise, the maximal displacement along the treadmill did not vary over conditions either (18.1 ± 1.1 cm; *F*(5, 55) = 1.38, *p* = 0.247, *ES* = 0.111; Fig 3A). The DFA scaling exponents α varied systematically over conditions for ST (*F*(3.47, 38.18) = 56.16, *p* < 0.001, *ES* = 0.836) and SL (*F*(4.61, 50.69) = 20.83, *p* < 0.001, *ES* = 0.654). Post-hoc analyses showed that α differed significantly between nearly all conditions for ST time series (all but ISO vs. α_0.2_ and α_0.5_ vs. α_0.6_ conditions) and SL time series (all but UC vs. α_0.9_, ISO vs. α_0.2_ and α_0.5_ vs. α_0.6_ conditions).

### Coupling strength between ST and SL

For the coupling between ST and SL, a significant effect of Condition was observed (*F*(5, 55) = 16.24, *p* < 0.001, *ES* = 0.596; Fig 3B). According to the post-hoc analyses, the correlation for the persistent metronome condition with α ≈ 0.9 was significantly higher than all other conditions. The correlation for the other persistent metronome condition with α ≈ 0.6 was significantly higher than all other conditions, except for the pacing condition with uncorrelated interbeat intervals (with α ≈ 0.5). Finally, the correlation coefficients for the uncued condition and the isochronous metronome condition were significantly lower than all other conditions.

### Surrogate analysis

Phase-randomization and cross-correlated phase-randomization yielded ST and SL surrogates with–by definition–DFA scaling exponents α that were indistinguishable from those derived for original ST and SL time series (Fig 2, panels B and C), for all six conditions. As expected, cross-correlated phase-randomized SS surrogates, which were obtained by taking the ratio of phase-randomized SL surrogates over phase-randomized ST surrogates (for which the phases were randomized simultaneously in exactly the same way), yielded DFA scaling exponents α that were similar to those observed for original SS time series, for all conditions alike (i.e., compare the black diamonds to the gray circles in Fig 2A). In contrast, phase-randomized SS surrogates showed DFA scaling exponents α that were markedly different from those derived from the original SS time series in all conditions (i.e., compare the white squares to the gray circles in Fig 2A), except for isochronous pacing and for the anti-persistent metronome condition.

## Discussion

In the present study we reanalyzed the data of Marmelat et al. [11] to gain more insight into the dynamics of stride times, stride lengths and stride speeds for treadmill walking paced by isochronous and non-isochronous metronomes with anti-persistent, uncorrelated and persistent correlational structures in interbeat intervals. The analysis was complemented by an examination of the coupling between stride times and stride lengths over the different pacing conditions. Of particular interest was the question how participants coupled their gait to a persistent metronome without wandering off the treadmill. Answering this question allows evaluating the premise that by using persistent metronomes in gait rehabilitation stride-to-stride dynamics would more closely resemble that of self-paced walking healthy participants [8, 10, 11, 14, 20].

First, we expected anti-persistence for stride speeds in all conditions to prevent wandering off the treadmill. We indeed found anti-persistence in stride-speed fluctuations for all conditions (Fig 2A), accompanied by maximal absolute fore-after displacements along the treadmill that fell well within the treadmill boundaries (Fig 3A). Together, these results confirm that participants tightly regulated stride speeds in all conditions, thereby extending the related findings of Dingwell and Cusumano for self-paced treadmill walking [3] and those of Terrier and Dériaz for isochronously paced treadmill walking [18] to non-isochronously paced treadmill walking.

Second, we found the expected increase in α for both stride times and stride lengths from anti-persistence to persistence over the four non-isochronous metronome conditions (i.e., α ≈ 0.2, α ≈ 0.5, α ≈ 0.6, and α ≈ 0.9; Fig 2, panels B and C). In combination with the observed anti-persistence in stride speeds for all conditions, this finding suggests that participants simultaneously controlled both stride times and stride lengths in such a way that deviations in either variable were cancelled out by concomitant changes in the other. This suggestion, first proposed by Dingwell and Cusumano ([3]; see also [18]), was corroborated by our observation that correlations between stride times and stride lengths were positive in all conditions (Fig 3B) and also by the results of the surrogate analysis, showing that only the DFA scaling exponents α of the cross-correlated phase-randomized surrogates were indistinguishable from the DFA scaling exponents α for original stride-speed time series in all conditions (Fig 2). The difference between phase-randomized surrogates and cross-correlated phase-randomized surrogates for stride-speed dynamics became most evident for the persistent metronome conditions and for the self-paced treadmill walking condition. For the latter condition, Dingwell and Cusumano [3] and Terrier and Dériaz [18] similarly observed that only cross-correlated phase-randomized surrogates successfully replicated stride-speed dynamics. This converging evidence strongly advocates that participants simultaneously controlled both stride times and stride lengths in order to limit stride-speed variations while walking on a treadmill. The coupling results (Fig 3B) revealed that this type of control became particularly manifest for the persistent metronome conditions, as will be discussed next.

Given that the inherent risk of wandering off the treadmill increases over the four non-isochronous metronome conditions (i.e., from α ≈ 0.2 to α ≈ 0.9), an increasingly stronger coupling between stride times and stride lengths was expected, with the highest correlation values for the persistent metronomes. Indeed, correlation values were significantly higher for the two persistent metronome conditions than for the other conditions, providing further evidence for the suggestion that participants simultaneously controlled both stride times and stride lengths, and particularly so in the persistent metronome conditions. The benefit of this type of control is that it allows for persistence to occur in stride times (as imposed by the persistent metronome conditions) while, by coupling stride-length deviations, limiting fore-after displacements along the treadmill, hence affording participants to maintain walking on the treadmill while coupling gait to the beat of a persistent metronome.

Finally, we would like to take our results to evaluate the use of persistent metronomes in gait rehabilitation, which is currently gaining prominence in gait rehabilitation research [8, 10, 11, 13, 14, 20]. The merit of this idea is difficult to establish at the moment because carry-over effects of persistent metronomes on stride-time dynamics have not been widely investigated. A notable exception in favor of retention is a recent study utilizing persistent visual metronomes for gait entrainment [14]. In another recent study, in which the clinical efficacy of persistent acoustic metronomes for changing the scaling exponent of stride times in Parkinson’s disease patients was examined, no transfer effects were observed [20]. Hence, given the absence of convincing empirical evidence in favor or against retention effects of walking paced by a persistent acoustic metronome, the merit of using persistent metronomes in gait rehabilitation is still an open question for future research.

This limitation notwithstanding, Marmelat et al. [11] observed qualitatively similar stride-time scaling exponents α for the uncued condition and the non-isochronous metronome condition with α ≈ 0.9, which led them to conclude that “the presence of long-range correlations in auditory cues enabled participants to maintain their ‘normal’, fractal gait pattern”. The current study also found qualitatively similar stride-length and stride-speed scaling exponents α for the uncued condition and the non-isochronous metronome condition with α ≈ 0.9 (Fig 2), which may be taken as additional evidence in support of their conclusion. However, the coupling strength between stride times and stride lengths deviated considerably between the two conditions (Fig 3B), which implies that the qualitatively similar correlational structure in stride-time, stride-length and stride-speed stride-to-stride fluctuations may have resulted from very different types of control. After all, these were the conditions with, respectively, the weakest and the strongest coupling between stride times and stride lengths (Fig 3B). A strong coupling indicates that stride-time deviations are accompanied by concomitant stride-length deviations in the same direction, a very tight type of control. Thus, whereas at first sight the use of persistent metronomes indeed evoked stride-time dynamics that better resembled that of self-paced walking healthy participants, in line with the premise, it likely resulted from a very tight regulation of stride speeds by strongly and positively coupling deviations in stride times to concomitant deviations in stride length–very different from self-paced treadmill walking healthy adults.

A recommendation for future studies would be to examine stride-to-stride fluctuations not only in terms of stride times, but to complement that with an assessment of stride-length and stride-speed dynamics as well as their interrelations. This may allow for testing the hypothesis that for longer treadmills a weaker coupling between stride times and stride lengths may be expected, given the extended maneuverability range along the treadmill and hence a reduced need for tightly regulating stride speeds. The same holds for overground walking. Another avenue for future research may be to test whether greater deviations in stride times (or stride lengths) during treadmill walking are accompanied by a stronger coupling between stride times and stride lengths. This might be addressed experimentally in various ways, for example, by comparing the coupling strength between patients with gait pathologies and healthy controls (a stronger coupling is expected for patients because they typically exhibit increased stride-time variability; [37]). Another option would be to evoke stride-time deviations with auditory stimuli, such as with the non-isochronous persistent metronome conditions in the current experiment, or stride-length deviations with a sequence of irregular stepping targets (e.g., [38]). Alternatively, rhythm perturbations could be applied in a sequence of isochronous interbeat intervals or inter-stepping target distances [39–41], which likely results in a similar positive correlation between perturbation-evoked step-length and step-time adjustments. In fact, Bank et al. [39] already observed that a longer step length was accompanied by a longer step time in perturbation-evoked long-step responses and vice versa for perturbation-evoked short-step responses (cf., Fig 3 in [39]). A concomitant adjustment of stride lengths and stride times seems an effective strategy to limit fore-after displacement along the treadmill, particularly in the presence of large variations in stride times and/or stride lengths: the stronger the coupling between the two, the more effective the strategy.

## Conclusions

Participants are able to follow the beat of a non-isochronous persistent metronome without falling off the treadmill by coupling stride-length deviations to the stride-time deviations evoked by the metronome, thus limiting variation in stride speeds and hence displacement along the treadmill. This coupling increased strongly from self-paced treadmill walking to paced treadmill walking with non-isochronous persistent metronomes. Although the correlational structure of stride times, stride lengths and stride speeds was similar for self-paced walking and persistent metronome conditions, the coupling results challenge the premise that stride-to-stride dynamics would better resemble that of self-paced walking healthy participants when pathological gait is paced by persistent metronomes. For an encompassing assessment of gait dynamics, we suggest that future studies complement the conventional stride-time time series with stride-length and stride-speed time series, and also analyze their interrelation in addition to the correlational structure of either one in isolation.

## Supporting Information

S1 Dataset Containing all stride-time, stride-length and stride-speed time series. (ZIP)
